# Effect of UV and Gamma Irradiation Sterilization Processes in the Properties of Different Polymeric Nanoparticles for Biomedical Applications

**DOI:** 10.3390/ma13051090

**Published:** 2020-03-01

**Authors:** Y. S. Tapia-Guerrero, M. L. Del Prado-Audelo, F. V. Borbolla-Jiménez, D. M. Giraldo Gomez, I. García-Aguirre, C. A. Colín-Castro, J. A. Morales-González, G. Leyva-Gómez, J. J. Magaña

**Affiliations:** 1 Sección de Estudios de Posgrado e Investigación, Escuela Superior de Medicina, Instituto Politécnico Nacional, Plan de San Luis y Díaz Mirón, Ciudad de México 11340, Mexico; yessicasarai@gmail.com (Y.S.T.-G.); jmorales101@yahoo.com.mx (J.A.M.-G.); 2 Laboratorio de Medicina Genómica, Departamento de Genética (CENIAQ), Instituto Nacional de Rehabilitación-Luis Guillermo Ibarra Ibarra (INR-LGII), Ciudad de México 14389, Mexico; fvbj@hotmail.com (F.V.B.-J.); usedat@gmail.com (C.A.C.-C.); 3 Laboratorio de Tecnología Farmacéutica, Facultad de Estudios Superiores Cuautitlán, Universidad Nacional Autónoma de México, Cuautitlán Izcalli, Edo. de México 54740, Mexico; luisa.delpradoa@gmail.com; 4 Departamento de Farmacia, Facultad de Química, Universidad Nacional Autónoma de México, Ciudad Universitaria, Ciudad de México 04510, Mexico; 5 Programa de Ciencias Biomédicas, Facultad de Medicina, Universidad Nacional Autónoma de México, Ciudad de México 04510, Mexico; 6 Departamento de Biología Celular y Tisular, Facultad de Medicina, Universidad Nacional Autónoma de México (UNAM), Edificio “A” 3er piso, Circuito Interior, Avenida Universidad 3000, Ciudad Universitaria, Coyoacán, Ciudad de México 04510, Mexico; 7 Unidad de Microscopía, Facultad de Medicina, Universidad Nacional Autónoma de México (UNAM), Edificio “A” planta baja, Circuito Interior, Avenida Universidad 3000, Ciudad Universitaria, Coyoacán, Ciudad de México 04510, Mexico; 8 Departamento de Genética y Biología Molecular, Centro de Investigación y de Estudios Avanzados (CINVESTAV-IPN), Ciudad de México 07360, Mexico; 9 Departamento de Infectología, (CENIAQ), Instituto Nacional de Rehabilitación-Luis Guillermo Ibarra Ibarra, Ciudad de México (CDMX) 14389, Mexico; 10 Escuela de Ingeniería, Departamento de Biotecnología, Instituto Tecnológico y de Estudios Superiores de Monterrey-Campus, Ciudad de México 14380, Mexico

**Keywords:** nanoparticles, sterilization, ultraviolet radiation, gamma radiation, poly (d,l-lactide-*co*-glycolide) acid, poly-(ε-caprolactone)

## Abstract

The sterilization processes of nanoparticles (NP) by autoclaving and filtration are two of the most utilized methods in the pharmaceutical industry but are not always a viable option. For this reason, the search for alternative options such as UV and gamma radiation is of interest. In this work, we evaluated both types of sterilization on two types of NP in solid state widely employed in the literature for biomedical applications, poly-(ε-caprolactone) and poly(d,l-lactide-*co*-glycolide) acid NP stabilized with polyvinyl alcohol. Physicochemical properties and cell viability were studied pre- and post-sterilization. The efficiency of irradiation sterilization was performed by a test of sterility using 1 × 10^8^ CFU/mL of *Escherichia coli*, *Staphylococcus aureus*, and *Candida albicans*. Microbiological monitoring revealed that both methods were sufficient for sterilization. After the UV irradiation sterilization (100 µJ/cm^2^), no substantial changes were observed in the physicochemical properties of the NP or in the interaction or morphology of human glial cells, though 5 and 10 kGy of gamma irradiation showed slight changes of NP size as well as a decrease in cell viability (from 100 µg/mL of NP). At 5 kGy of radiation doses, the presence of trehalose as cryoprotectant reduces the cell damage with high concentrations of NP, but this did not occur at 10 kGy. Therefore, these methods could be highly effective and low-processing-time options for sterilizing NP for medical purposes. However, we suggest validating each NP system because these generally are of different polymer-composition systems.

## 1. Introduction

Polymeric nanoparticles (NP) have shown to be promising drug-delivery systems for use in pharmaceutical and biomedical applications. NP have been considered as solid colloidal particles ranging in size from 1 to 1000 nm [1,2] and consisting of macromolecular materials in which the cargo (drugs or biomolecules) is dissolved, entrapped, or encapsulated, or to which the active principle is adsorbed or attached [3]. They can incorporate multiple drugs and molecules into different tissues with high biocompatibility, biodegradability, low toxicity, and prolongation of release times [4,5]. Among others, poly-(ε-caprolactone) (PCL) and poly-(d,l-lactic-*co*-glycolic) acid (PLGA) are two of the most effective matrix agents for nanoparticles [6], that can be manufactured with different shapes and sizes with high reproducibility [7] and belong to the family of US Federal Drug Administration (FDA)-approved degradable polymers [8,9]. Depending on the material with which they are designed and the resulting physical and chemical characteristics, multiple nanoparticulate systems have been developed to be used as pharmacological vehicles or gene therapy vectors for several tissues such as central nervous system (CNS) delivery that can be made up of hydrophobic polymers such as poly(lactic acid) (PLA), PLGA, and PCL [4].

The usefulness of these NP formulations and the medical implications for their in vivo administration require sterile manufacturing conditions [10]. According to the United States Pharmacopeia (USP), NP formulations must be sterile; however, studies aiming at identifying new NP sterilization conditions are currently insufficient. In this regard, some sterilization methods have been applied, including heat, chemical methods, filtration, and ionizing sterilization. Nevertheless, for polymeric NP, some of these results, though efficient, cause alterations in the physicochemical properties [11]. Masson et al. analyzed heat sterilization, gamma radiation, and filtration in a PCL-based NP system with different surfactants, finding that only filtration did not affect the physical properties of the system. However, this required NP sizes of less than 220 nm in non-viscous fluids [12].

These findings demonstrated that heat sterilization resulted in agglomeration and color change, proving multiple structural modifications. Concerning gamma radiation, sterilization with 25 kGy increased the molecular weight of polymers in NP due to the formation of radicals and crosslinking between polymeric chains and even between surfactant and polymer chains. A similar strategy of analysis was performed in a PLGA (52:48) NP system [13]. After gamma-irradiation sterilization (25 kGy), no statistically significant differences were observed in mean particle size and Z-potential; however, the polydispersity index (PDI) increased notably. Obtaining homogenous dispersions was complicated, and the drug release was influenced negatively in sterilized NP [13]. On the other hand, Shahabi et al. used a PLGA (80:20)-PEG/hydroxyapatite composite to analyze the impact of different doses of gamma radiation, confirming a significant decrease in polymer molecular weight due to chain scission [14].

In 2005, Bozdag et.al., evaluated the effect of the gamma-irradiation sterilization on the characteristics of ciprofloxacin HCl-loaded poly(d,l-lactide-glycolide) nanoparticles [13]. The obtained results showed that the drug release was influenced negatively after gamma irradiation. In addition to these results, the effect of the nanoparticles against microorganisms was adequate. Similarly, 2006, Memisoglu-Bilensoy E. et al. elaborated cyclodextrin nanoparticles and evaluated the modification of several properties, including the drug entrapment and delivery, due to different sterilization process [11]. They demonstrated that the changes observed after gamma irradiation sterilization were not significant compared with the changes observed after autoclaving and filtration sterilization. Previous reports have described that high levels of gamma irradiation can alter the release profile of drugs; however, this depends on the constitution of both the polymer and the surfactants of each nanoparticulate system [15,16,17]. Therefore, although gamma-ray irradiation would provide effective NP sterilization, the physicochemical effects on NP systems could be questionable. For these reasons, other promising and rapid sterilization strategies have begun to be applied. In the case of ultraviolet (UV) light sterilization, there are few works on the application in NP. In nanotube and metal particle systems, UV sterilization was employed and proved to be effective for eliminating microorganisms, and no adverse changes were observed [18,19]. Overall, the various methods analyzed possess disadvantages in the NP formulations. Thus, it is necessary to find better sterilization conditions that do not modify the physicochemical properties of the NP. Even the sterilization efficacy of low doses of gamma irradiation (8 and 13 kGy) has been demonstrated [20] in the presence of some protective sugars such as glucose and mannitol at 5% in chitosan hydrogel-NP. Usually, the presence of cryoprotective agents is in considerable quantities in the final batches of NP, so it is reasonable to consider an additional effect of that excipient.

Therefore, in this study, we analyzed the effect of two sterilization methods based on low gamma irradiation and UV light on two kinds of nanoparticles widely described in the literature for medical applications (PCL and PLGA), with the presence of PVA as surfactant and a cryoprotective agent. The sterilization process was in solid state. A physicochemical analysis was performed before and after sterilization as well as a cell viability test in a neuronal cell line to determine the possible effects derived from gamma and UV irradiation. Under the analysis conditions, UV light proved to be safe and effective, while gamma irradiation slightly modified physical parameters and tended to increase cell death at high concentrations of NP.

## 2. Materials and Methods 

### 2.1. Materials

For the elaboration of NP, we used poly-ε-caprolactone (Mn 14,000 g/mol), poly(d,l-lactide-*co*-glycolide) (Mn 30,000-60,000 g/mol), Mowiol 4-88 (Polyvinyl alcohol 4-88), and D-(+)-trehalose dihydrate, which were supplied by Sigma-Aldrich^®^ (Merck KGaA, Dramstadt, Germany), whereas ethyl acetate was purchased from Spectrum^®^ (Spectrum Laboratory Products, NJ, USA). Mannitol was obtained from Central de Drogas, S.A. de C.V. (Mexico City, Mexico). For biological tests, we utilized Dulbecco’s modified Eagle’s medium (DMEM), penicillin-streptomycin, trypsin-EDTA, PBS 1X, and sodium pyruvate, which were obtained from Gibco^®^/Life Technologies (ThermoFisher Scientific, MA, USA); fetal bovine serum (FBS) was supplied by Biowest^®^ (Nuaillé, France), trypan blue and paraformaldehyde P6148 were supplied by Sigma-Aldrich^®^ (Merck KGaA, Dramstadt, Germany), and the Cell Proliferation Kit I (MTT) was purchased from Roche^®^ (Roche Diagnostics GmbH, Mannheim, Germany).

### 2.2. Preparation of Nanoparticle Systems

PCL/PVA NP and PLGA/PVA NP were obtained through the emulsification-diffusion method as previously reported [15]. Briefly, ethyl acetate was mixed with bi-distilled water (QGARD00R1, Merck Millipore^®^, Dramstadt, Germany), in a 1:1 ratio, in order to produce saturated phases (organic saturated phase—OSP, and aqueous saturated phase—ASP). For PCL/PVA NP, 400 mg of PCL was dissolved in OSP to obtain a 2% (*w*/*v*) solution. The mixture was added to PVA in ASP (5% *w*/*v*), and then it was emulsified by a high-speed homogenizer (Ultra Turrax T18 IKA^®^; Wilmington, NC, USA) at 11,000 rpm for 10 min at room temperature. Then, the diffusion medium (water at the same temperature) was added. After that, the organic solvent was evaporated by rotary vacuum (Heidolph^®^ Instruments GmbH & Co., KG, Schwabach, Germany). The resulting NP suspension was centrifuged only once at 11,000 rpm for 90 min at 4 °C (model 5804 R Centrifuge; Eppendorf^®^, Hamburg, Germany). Finally, the pellet was resuspended in distilled water. For the next system, PLGA/PVA NP were prepared with the same procedure except for that the PLGA was dissolved in OSP at 1% (*w*/*v*), and the solution of PVA was used at 2.7% (W/V) in ASP. Formulations were frozen and lyophilized (49 °C, 0.05 mBar, and 24 h). For NP PCL/PVA, 20% of D-(+)-trehalose was dissolved in 70 mg/mL of PCL/PVA NP, whereas 15% of mannitol was dissolved in 56.45 mg/mL of PLGA/PVA NP. The NP were first frozen at −20 °C in a Thermo Scientific ultrafreezer for 18 h, and then, the frozen systems was lyophilized at −45 °C for 2 h, at ramp 1 °C/min as in the previous report by Abdelwahed W et al. [21].

### 2.3. Sterilization Methods

Both NP systems were sterilized by two different methods, UV and gamma irradiation, for evaluating the possible physicochemical changes in NP after their processing. For UV irradiation, lyophilized NP (1 mg) and NP in an aqueous dispersion state (1 mg/mL) with a viscosity of 110 cP (viscous medium), were placed in 1.5 mL microcentrifuge tubes (Eppendorf tubes 3810X; Eppendorf, Hamburg, Germany) and sterilized by UV radiation during different exposure times (0.5, 1.0, 1.5, 2, 2.5, and 3 h) by means of a UVC 500 ultraviolet crosslinker (Amersham Biosciences^®^, UA) with a dose of 100 µJ/cm^2^, whereas for gamma irradiation, NP filling glass vials (20 × 55 mm) were packed in double sterilization pouches and were irradiated under air, gamma dose of 5 and 10 kGy at dose rate 125 Gy/min, using a Gamma beam 651PT irradiator with a ^60^Co gamma-ray source (AECL^®^, Atomic Energy of Canada Ltd., Ottawa, Canada). Both procedures were performed at room temperature. These experiments were repeated at least three times.

### 2.4. Microbiological Evaluation

After the preparation of the NP systems, these were dispersed (1 mg/mL) by means of mechanical agitation, and then 100 µL of NP were inoculated with radial diffusion on the surface of the culture media. The sheep blood agar 5% plates and MacConkey agar plates were for bacteria and coliform counts, while the Saboroud dextrose agar plates were for fungal count and were incubated at 35 °C and analyzed each 24 h for 7 days. Microorganism count was reported as the number of colony-forming units (CFU/mL). For surviving microorganism identification, the Gram staining was carried out in order to classify them according to the characteristics conferred by the walls of the bacteria. For a detailed analysis, Gram-negative (GN) bacilli were tested by indole and oxidase reaction. Meanwhile, for Gram-positive (GP) cocci, catalase and coagulase tests were conducted. For a complete identification of microorganisms, the VITEK 2 System (bioMérieux, Marcy d’Etoile, France) was employed.

On the other hand, the efficacy of the sterilization process was assessed by testing the sterility of the samples contaminated before the irradiation with a suspension of *Escherichia coli* ATCC 25922, *Staphylococcus aureus* ATCC 29213, and *Candida albicans* ATCC 10231. Concentrations of 1 × 10^8^ CFU/mL of the strains were analyzed in each vial containing each one of polymeric NP, and 0.5 mL of inoculum was added in 0.5 mL of NP dispersion. Prior to irradiation, the vials were sealed with rubber stoppers. After irradiation, the aliquots were inoculated onto the different agar plates and biochemical probes, previously described. The surviving microorganisms were counted after 24 h incubation at 35 °C for 7 days and were reported as the number of colony-forming units (CFU/mL).

### 2.5. Physicochemical Characterization

#### 2.5.1. Particle Size and Zeta-Potential Measurement

Particle size, distribution (referred as polydispersity index, PDI), and zeta-potential were determined using a Malvern Zetasizer Nano (Malvern Instruments model ZS90; Malvern, UK). Particle size and PDI were measured by dynamic light scattering (DLS), while zeta potential was measured by velocimetry laser doppler (VLS). Measurements were performed after the freeze-drying process at 25 °C. For physicochemical evaluation, lyophilized NP systems were re-suspended at 1 mg/mL through mechanical agitation at 3000 rpm for 1 h (Model MS 3 digital, IKA Works Inc., USA). Each value resulted from determinations in triplicate. Thus, the results are reported in terms of average ± standard deviation (SD).

#### 2.5.2. Scanning Electron Microscopy (SEM)

The morphology of the lyophilized NP was inspected by means of scanning electron microscopy (SEM) using a field-emission scanning electron microscope (Model JSM-7600F; Jeol Company, Japan). Prior to assessment, samples were prepared on cover glass and coated with gold, under an argon atmosphere, by means of an ion sputter coater (Model JFC-1100, Jeol Company Japan).

#### 2.5.3. Fourier Transform-InfraRed (FTIR) Spectroscopy

To evaluate the chemical interactions after the sterilization process, the freeze-dried NP and the reagents were analyzed by Fourier transform infrared (FITR) spectroscopy using a FTIR Nicolet 6700 spectroscope (Thermo Scientific, Waltham, MA, USA). The scanning range was 4000–400 cm^−1^, and the resolution was 2 cm^−1^.

#### 2.5.4. Thermogravimetric Analysis (TGA)

The thermal stability of the NP, with and without the sterilization process, was evaluated by TGA. Briefly, lyophilized samples of about 5 mg were tested in a Hi-Res TGA 2950 Thermogravimeter Analyzer (TA Instruments, Inc., DE, USA). Tests were carried out under a nitrogen atmosphere, from room temperature to 500 °C and at a heating rate of 10 °C/min.

#### 2.5.5. Differential Scanning Calorimetry (DSC)

The evaluation of NP thermal properties was performed by DSC. Lyophilized samples of NP before and after the sterilization process were tested in hermetic aluminum cells by means of a DSC 2910 (Modulated TA Instruments, Inc., DE, USA) under a nitrogen atmosphere, from room temperature to 250 °C and at a heating rate of 10 °C/min.

### 2.6. Biological Characterization

#### 2.6.1. Cell culture

To analyze the effect of sterilization methods in cell culture, we selected a human central nervous system (CNS) cell line denominated Müller glial (MIO-M1 cell line). Cells were kindly provided by Dr. Arturo Ortega from CINVESTAV-IPN, Mexico. Cells were maintained as a monolayer culture in DMEM and supplemented with 10% FBS and 1% penicillin/streptomycin, at 37 °C and 5% CO_2_ under a fully humidified atmosphere. The cell-culture medium was refreshed every 48 h. When the cells approached 80% confluence, they were trypsinized and sub-cultured in the growth medium and used for the viability test.

#### 2.6.2. Cell Viability Assay

The in vitro cytotoxicity of the NP, before and after the sterilization process, was determined using the Cell Proliferation Kit I (Roche Diagnostics GmbH, Mannheim, Germany) assay with the MIO-M1 cell line. Briefly, cells were seeded on a 96-well plate 24 h prior to the assay. The cells were then incubated with fresh medium (150 µL), including different concentrations of non-sterilized NP and sterilized NP (UV, 5 and 10 kGy, concentrations: 10, 20, 30, 60, 80, 100, 150, and 200 µg/mL) during 24 h (6000 cells/well), 48 h (4000 cells/well), and 72 h (1800 cells/well). Cells incubated with solely DMEM were referred to as untreated cells, and a dead control with 0.1 M of H_2_O_2_. Then, the cells were washed twice with PBS 1X. After that, 10 µL of the 3-(4,5-DiMethylThiazol-2-yl)-2,5-diphenyl tetrazolium bromide (MTT) solution (5 mg/mL) was added to each well, and the plates were incubated at 37 °C during 4 h in a humidified atmosphere. After that, the solubilization solution for each well was added and incubated overnight to solubilize the salt crystals formed. The formazan product was spectrophotometrically quantified using a Synergy HTX multi-mode microplate reader (BioTek^®^, Vermont, USA) at 570 nm. All experiments were arranged in triplicate for statistical analysis. Results were expressed as mean ± SD.

#### 2.6.3. Immunofluorescence and Confocal Microscopy Analysis

Immunostaining and CLSM analyses were carried out following standard techniques. Briefly, MIO-M1 cells (12,000 cells) were seeded in tissue culture dishes containing coverslips and incubated during 24 h. Cells were washed twice with PBS, and treatment with PCL/PVA or PLGA/PVA NP was added at a concentration of 80 µg/mL or 200 µg/mL without sterilization, UV irradiation, or 5 or 10 kGy gamma irradiation during 48 h. After the period of incubation, cells were washed twice with PBS and fixed with 4% of paraformaldehyde in PBS for 20 min. After fixation, cells were washed twice and permeabilized with 2% triton X-100 in PBS for 5 min. Then, they were washed twice and blocked with 0.5% gelatin and 1.5% FBS in PBS for 20 min. After that, the cells were labelled with phalloidin primary antibody (Jackson ImmunoResearch Laboratories), and washed twice with PBS. Coverslips were mounted in slides with VectaShield/DAPI (Vectorlabs^®^) and sealed. Images were acquired with the confocal laser system TCS-SP5 (Leica Microsystems, Germany).

## 3. Results and Discussion

### 3.1. NP Manufacture and Physicochemical Characteristics

**Figure 1:**
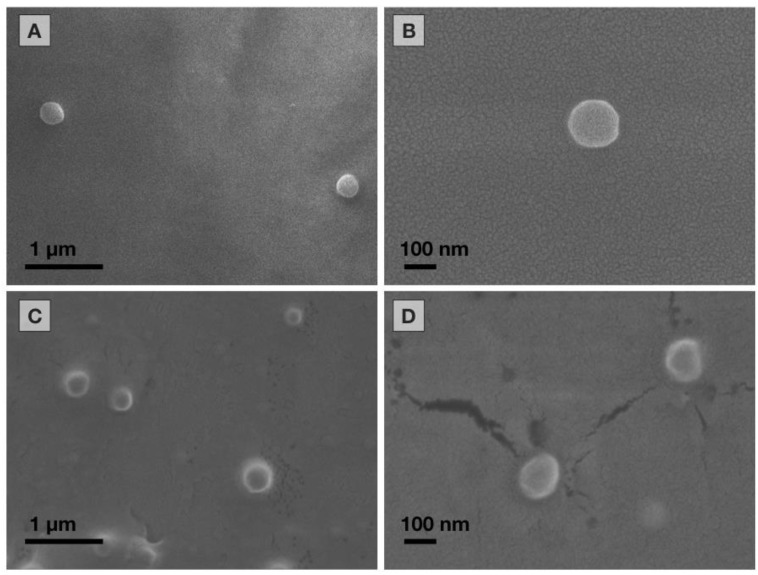
Morphology of nanoparticles by scanning electron microscopy. (**A**) PCL/PVA 25,000×, (**B**) PCL/PVA 100,000×, (**C**) PLGA/PVA nanoparticles (NP) 25,000×, and (**D**) PLGA/PVA NP 100,000×.

The NP systems were obtained by the emulsification-diffusion method with an average size of 228.8 ± 11.60 and 243.1 ± 3.06 nm for PCP/PVA NP and PLGA/PVA NP, respectively, and with PDI values lower than 0.1 (Table 1), which represents a homogeneous particle size. Both characteristics are compatible for medical purposes, considering that the use of NP as drug carriers can achieve a range of up to 500 nm. These features correspond to systems with a viable size in order to be able to permeate different biological tissues. Some studies have even demonstrated that a size of 420 nm has the ability to cross the blood–brain barrier (BBB) [22], which can increase with surface modifications through the functionalization or coating by different molecules or surfactants [23]. These features are compatible for medical purposes and could be used as drug nanocarriers. In addition, their homogeneous size distribution allows having control over the biological effect of the possible drugs to be transported. Zeta potential values for PCL/PVA NP and PLGA/PVA NP were −14.5 ± 1.76 and −17.0 ± 0.17 mV, respectively. These values are in agreement for colloidal systems with an adequate stabilization in aqueous dispersion [17,24]. In correlation with Zetasizer measurements, SEM observations revealed particles with a regular rounded-surface shape; the dimensions in terms of the longer axis are between 200 and 250 nm with no agglomeration (Figure 1). These NP optimized the relationship between surface and volume, thus providing adequate carriers with effective capillary mobilization for drug delivery [25,26].

### 3.2. Microbiological Evaluation Before and After Sterilization

Interestingly, the sizes found in our systems are not susceptible to sterilization by filtration. It has been widely reported that a sterile filtration can be applied to polymeric nanospheres if the mean diameter is below 220 nm, and even particles around of 200 nm present physical limitations like a poor recovery of NP [12,27,28]. Therefore, we proposed the use of two sterilization methods based on UV light and low doses of gamma irradiation. The selection of these methods was based on their relatively low cost and their easy accessibility. UV light is an electromagnetic radiation with germicidal properties at 250–270 nm (UVC region) that has been widely used in the medical and food areas [29]. The high energy associated with a short wavelength is absorbed by cellular nucleic acid, breaking molecular bonds in DNA within microorganisms, creating new bonds among nucleotides, and producing thymine dimers that can kill or disable bacteria, viruses, and protozoa [30]. UV light provides rapid and effective inactivation free of chemicals or corrosive substances, does not produce carcinogenic substances, and is effective against a wide range of bacteria [29]. On the other hand, the effect of gamma irradiation as a sterilizing process has been widely described, its effectiveness has been well recognized, and one of the best pieces of evidence comprises the industrial volumes processed in different countries, both in the food and pharmaceutical sectors [31]. However, multiple studies have reported diverse morphological and chemical alterations with irradiation above 15 kGy [13,14]; hence, it is important to assess lower doses to determine their sterilization effect and the effect at the level of the nanoparticle.

Baseline microbiological evaluation demonstrated that the identification of the Gram burden of non-irradiated samples was ∼102 CFU/mL in PCL/PVA; in addition, we found the presence of 5 CFU/mL of *Aspergillus niger* in PLGA/PVA NP. Then, we evaluated the irradiation resistance of microorganisms found in both NP systems after these sterilization methods, in which we were unable to identify the growth of any microorganism; the latter suggested that both methods of sterilization possess the elimination capacity of common microorganisms. For the validation of the sterilization process, both of the NP systems samples were inoculated before irradiation with an independent suspension of *E. coli* and *S. aureus*, as well as of *C. albicans.* Then, the samples were sterilized by either irradiation method, and the non-irradiated samples were used as control. As demonstrated by the microbiological assay, sterilization of the samples was achieved effectively with each treatment independent of the type of irradiation. For UV irradiation, we selected 2 h of treatment, since it was the minimum sterilization time in NP in solid state to eliminate 100% of all microorganisms after performing a response curve with conventional equipment that could be easily reproduced (Figure S1). At least in these systems, irradiation with UV with a dose of 100 µJ/cm^2^ for 2 h and gamma irradiation at low doses was sufficient to obtain results that allowed suitable sterilization for the elimination of different microorganisms. It is important to mention that the sterilization processes were carried out in small tubes with solid-state samples. For other conditions such as liquid state or larger samples, the necessary conditions should be adapted, since UV light can have a limited penetration range. We found that in aqueous dispersion (1 mg/mL) of NP with a higher viscosity, longer exposure time to UV irradiation was necessary (Figure S1). In addition, further studies are required to validate these results in shorter UV wavelengths, as the system that we used keeps the wavelength predefined.

### 3.3. Influence of Sterilization on the Physical Characteristics of Nanoparticles

With the aim to evaluate the effect of the sterilization process on NP properties, the freeze-dried samples were analyzed by DLS. Table 1 presents the comparison of average particle size, PDI value, and zeta potential, before and after the sterilization process. For both PCL/PVA and PLGA/PVA NP, there was no difference after sterilization for 2 h by means of the ultraviolet irradiation. Even so, the values of PDI and zeta potential did not exhibit a significant change. SEM observations revealed particles without changes between non-sterilized NP and UV irradiation NP (Figure S2A,B,E,F).

Exposure of both NP systems to gamma rays showed certain effects on average particle size, PDI, and zeta-potential values, as demonstrated in Table 1. We observed a slight tendency to decrease in particle size and zeta potential, and an increase in the value of the PDI with an increase in the dose of gamma radiation. SEM analysis showed a slight decrease in NP size after gamma irradiation (Figure S2C,D,G,H). This effect can be due to the known phenomenon of “long-chain excision” [32], possibly in those of PVA that are exposed to the external environment. Radiation in an aqueous medium produces the primary reactive species •OH, e_aq_, and H• [33,34], which leads to the chain scission of PVA with a consequent decrease in molecular weight. A similar effect is observed in the particle size for the PLGA/PVA NP system with gamma radiation; however, the changes in zeta potential are not notable. The difference in zeta potential for PCL/PVA NP could be due to the interactions of PVA on the NP surface and the type of anchoring to the matrix polymer. Even with these slight modifications, it is important to note that, for general medical purposes, the observed modifications were not relevant.

The morphology of both irradiated systems was analyzed by SEM. The samples analyzed maintained their morphology (Figure S2). Possible morphological changes were not evidenced by SEM due to the low resolution for distinguishing changes in the PVA chains. However, the effect on the NP is imperceptible. It is not possible to observe agglomeration; therefore, the possible changes do not appear to affect the properties of the NP.

### 3.4. Influence of Sterilization on the Chemical Characteristics of NP

#### 3.4.1. Fourier-Transform Infrared Spectroscopy (FTIR)

Diverse sterilization techniques have been related to inducing changes in the characteristics of materials and properties, depending on the physicochemical nature of the polymers [35,36]. Thus, the intensive chemical evaluation after sterilization method is necessary as the doses and times of sterilization application vary according to the material. To determine whether the modifications found in our systems gave rise to chemical alterations, we decided to analyze the structural evaluation of the excipients and the NP initially, before and after the sterilization process, through FTIR analysis.

**Figure 2:**
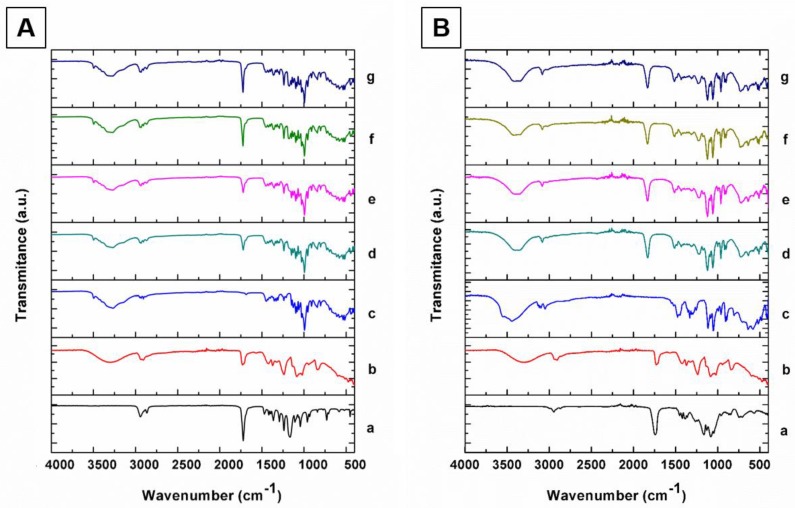
(**A**) Spectra of the PCL/PVA NP system; (a) PCL, (b) PVA, (c) trehalose, (d) non-sterilized PCL/PVA NP, (e) PCL/PVA NP after UV radiation, (f) PCL/PVA NP after 5 kGy of gamma radiation, and (g) PCL/PVA NP after 10 kGy of gamma radiation. (**B**) Spectra of the PLGA/PVA NP system; (a) PLGA, (b) PVA, (c) mannitol, (d) non-sterilized PLGA/PVA NP, (e) PLGA/PVA NP after UV radiation, (f) PLGA/PVA NP after 5 kGy of gamma radiation, and e) PLGA/PVA NP after 10 kGy of gamma radiation.

Figure 2A depicts the PCL/PVA NP spectra, in which line (a) corresponds to the PCL spectrum. The bands presented at 2940 and 2866 cm^−1^ are attributed to asymmetrical and symmetrical C-H_2_ stretching, respectively. The band related to carbonyl stretching appears at 1740 cm^−1^; these results are in agreement with those of the literature [37]. Meanwhile, line (b) shows the PVA spectrum; the band at 3100–3600 cm^−1^ is attributed to the stretching vibration of O-H from the intermolecular and intramolecular hydrogen bonds. The band observed at 2840–3000 cm^−1^ is related to the C-H stretching of C-H bonds present in alkyl groups [38]. Finally, the band observed at 1726 cm^−1^ is associated with the C=O vibration. Trehalose spectra are presented at line (c) and exhibit different characteristic bands. O-H bond stretching is responsible for the 3479 and 3251 cm^−1^ bands [39,40]. The spectra of PCL/PVA NP system before the sterilization process correspond to line (d). The latter exhibited the characteristic peaks of PCL, PVA, and trehalose. As could be observed, there were not evident modifications after UV and gamma irradiation, as shown in lines (e), (f), and (g), respectively.

The PLGA/PVA NP spectra (Figure 2B) show the line (a) that represents the spectrum of PLGA, presenting bands at 2870–3000 cm^−1^, which, according to the literature, are associated with C-H, C-H_2_, and C-H_3_ stretching vibrations [41,42]. At 1421 cm^−1^, the band corresponding to C-H stretching in methyl groups was also found. Line (b) corresponds to the PVA spectrum (previously described). For mannitol (line c), the band could be observed that related to the O-H stretching vibration at 3270 cm^−1^, whereas C-H deformation vibrations could be observed between 1400 and 1200 cm^−1^ [43]. Line (d) depicts the spectra of the PLGA/PVA NP system before any sterilization procedure. The bands can be identified that correspond to PLGA, PVA, and mannitol. As in the case of PCL/PVA NP, there are no significant changes after UV and gamma irradiation. Interestingly, despite having found a slight decrease in particle size under gamma irradiation, it was not possible to detect apparent chemical modifications; these results suggest that both sterilization techniques could be applied for the systems manufactured in this research.

#### 3.4.2. Thermogravimetric Analysis (TGA)

**Figure 3:**
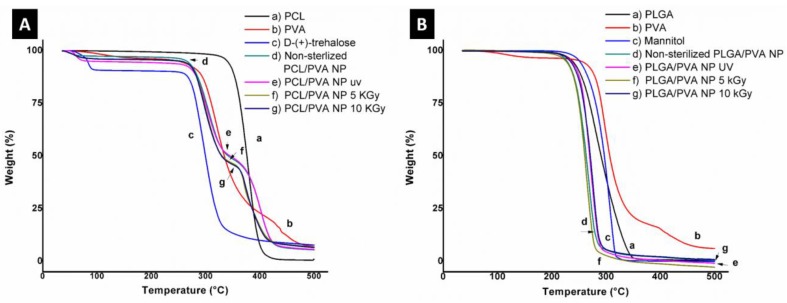
(**A**) Thermogram of the PCL/PVA NP system; (a) PCL, (b) PVA, (c) trehalose, (d) non-sterilized PCL/PVA NP, (e) PCL/PVA NP after UV radiation, (f) PCL/PVA NP after 5 kGy of gamma radiation, and (g) PCL/PVA NP after 10 kGy of gamma radiation. (**B**) Thermogram of the PLGA/PVA NP system; (a) PLGA, (b) PVA, (c) mannitol, (d) non-sterilized PLGA/PVA NP, (e) PLGA/PVA NP after UV radiation, (f) PLGA/PVA NP after 5 kGy of gamma radiation, and (g) PLGA/PVA NP after 10 kGy of gamma radiation.

To confirm the absence of chemical alterations, Figure 3 illustrates the TGA profile of both systems: PCL/PVA NP and PLGA/PVA NP. Figure 3A presents the thermogram of PCL/PVA NP. Line (a) corresponds to the PCL profile; only one weight-loss step could be identified. This step begins at 328 °C and ends at 417 °C, in agreement with that reported by Mohamed et al. and Persenaire et al. [44,45]. Two steps of decomposition can be observed for the line (b), which corresponds to PVA; the first of these steps is between 240 °C and 340 °C and second between 390 °C and 470 °C, which is in agreement with the literature [46,47,48]. Line (c) reveals two weight-loss stages in the thermal behavior of trehalose. The first weight loss, at 100 °C, corresponded to the dehydration of trehalose dehydrate into anhydrous trehalose [49] while the second stage was related to the degradation of the sample (250–330 °C). PCL/PVA NP before and after the sterilization process, (lines d, e, f, and g) exhibited the same profile. Briefly, after the water evaporation (100 °C), the thermograms showed two thermal events, the first from 260 to 335 °C, and the second between 335 and 430 °C. The first step is related to the PVA presence in the nanosystems. The latter thermal event occurred from 450 to 559 °C, presenting a total loss of 90%, approximately. The thermogram of PLGA/PVA NP is shown in Figure 3B. Line (a) presents one weight-loss step between 190 and 349 °C, corresponding to PLGA, as previously reported by Ávila et al. [50]. This degradation mechanism could be related to a random chain scission when the degradation begins and to a specific chain scission at the end of the mechanism [51]. The PVA thermogram demonstrated the same behavior as shown in PCL/PVA NP. For mannitol (line c), we were able to observe, as in PLGA, one step of degradation that begins at 197 °C and fends at 323 °C. PLGA/PVA NP, prior to the sterilization process (line d) exhibited similar behavior to PLGA and mannitol, but the NP presented lower degradation temperatures than the excipients. This may be due to the NP possessing a larger surface area, meaning higher reactivity than the component alone. It could be observed that, after UV and gamma irradiation (lines e, f, and g), the NP presented the same profile as NP before any sterilization treatment. These results suggested that both types of sterilization techniques did not affect the thermal characteristics of the NP.

#### 3.4.3. Differential Scanning Calorimetry (DSC)

**Figure 4:**
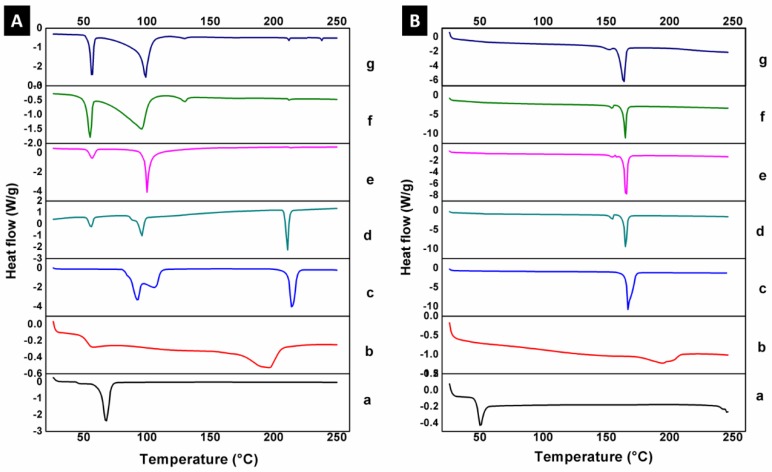
Differential scanning calorimetry of nanoparticles. (**A**) Thermogram of the PCL/PVA NP system; (a) PCL, (b) PVA, (c) trehalose, (d) non-sterilized PCL/PVA NP, (e) PCL/PVA NP after UV radiation, (f) PCL/PVA NP after 5 kGy of gamma radiation, and (g) PCL/PVA NP after 10 kGy of gamma radiation. (**B**) Thermogram of the PLGA/PVA NP system; (a) PLGA, (b) PVA, (c) mannitol, (d) non-sterilized PLGA/PVA NP, (e) PLGA/PVA NP after UV radiation, (f) PLGA/PVA NP after 5 kGy of gamma radiation, and (g) PLGA/PVA NP after 10 kGy of gamma radiation.

The thermal properties of NP by DSC are depicted in Figure 4 and described in Table 2. In agreement with the literature, PCL and PVA presented a melting temperature (Tm) of 67.5 and 195.5 °C, respectively. D-(+)-trehalose dehydrate demonstrated two important thermal events; the first at 100 °C represented dehydration of trehalose di-hydrate into anhydrous trehalose, while the second temperature illustrated a sharp endotherm located at 212 °C, corresponding to the melting. All of the PCL/PVA systems showed the two trehalose peaks as main signals, due to the proportion in which they are found with respect to the other components. In addition, a left displacement is observed for the PCL thermal event from 67.5 to 56 °C. Interestingly, a thermal event appears at 130 °C for PCL/PVA NP 5 kGy and PCL/PVA NP 10 kGy. This peak could be related to a degradation sub-product derived from trehalose induced by gamma radiation.

Likewise, the glass transition temperature of PLGA was recorded at 50.4 °C along with a second thermal event of decomposition at 241.4 °C. Mannitol exhibited a melting temperature of 167 °C. The PLGA/PVA systems (PLGA/PVA NP without treatment, PLGA/PVA NP after UV radiation, PLGA/PVA NP after 5 kGy of gamma radiation, and PLGA/PVA NP after 10 kGy gamma radiation) exhibited one main peak about 165 °C, attributed to a shift to the left of the first thermal event of the mannitol. This could correspond to a cleavage in the structure of mannitol and a decrease in the molecular weight, and, therefore, in the melting point.

### 3.5. Biological Characterization

**Figure 5:**
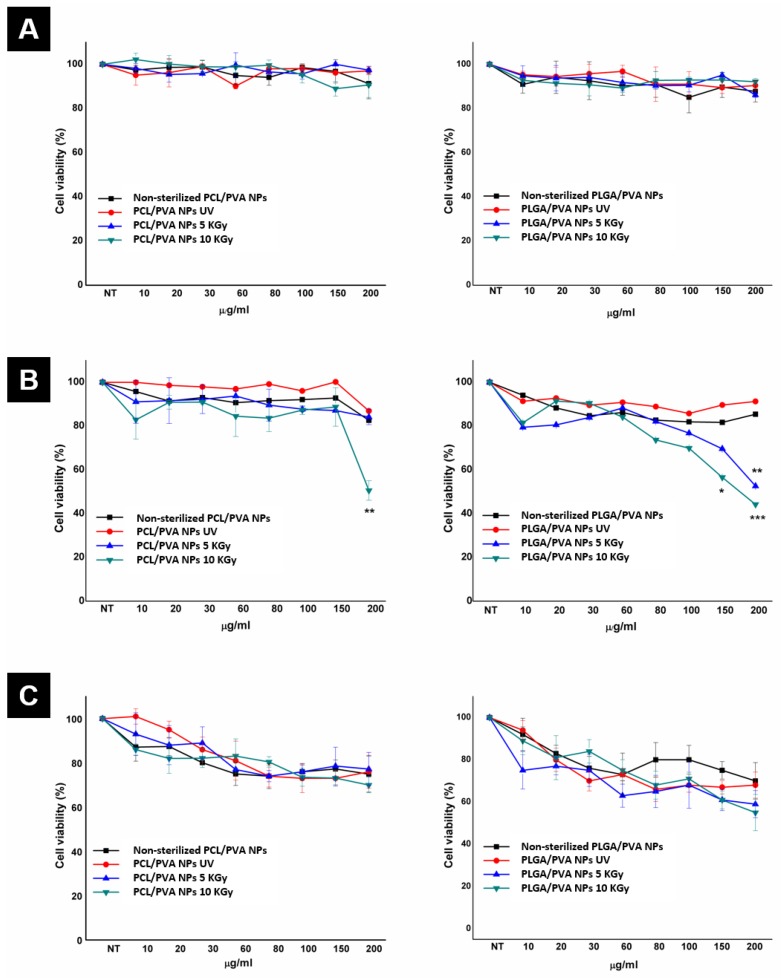
PCL/PVA NP and PLGA/PVA NP cell viability by MTT assay. Cells were treated for 24 h (**A**), 48 h (**B**), and 72 h (**C**) with 10-200 µg/mL of PCL/PVA NP and PLGA/PVA NP, respectively. UV irradiation (red lines), 5 KGy (blue lines) and 10KGy (green lines) gamma irradiation were compared with non-sterilized NP (black lines). Untreated cells were considered as a value of 100% viability. The results are expressed as the mean ± SD of three separate experiments. * *P* ≤ 0.1, ** *P* ≤ 0.01, and *** *P* ≤ 0.001 compared with non-treated cells (control).

Physicochemical modifications of NP induced by the sterilization process were evidenced with minor changes. However, we evaluated the influence of the sterilization on the cell viability of a human cell line of the CNS, due to the potential use of these NP as delivery-drug systems for crossing the BBB. We selected a glial human cell model (MIO-M1) for the role in which glial cells provide support and protection to neurons [52]. The viability of the MIO-M1 cells was evaluated by MTT assay in the presence of different concentrations, ranging from 10 to 200 µg/mL of both NP systems (Figure 5) and incubated for 24, 48, and 72 h, respectively, in order to detect cellular adverse effects after the sterilization process. Untreated cells with only medium were also included. At 24 h it was not possible to observe alterations on cell viability for both systems (Figure 5A). At 48 h, only a small number of dead cells were observed. For PCL/PVA NP all the experiments presented a percentage of cell viability between 100 and 80 %, with no statistical significance (Figure 5B). However, PCL/PVA NP at 10 kGy affected cell viability at a higher concentration (200 µg/mL). On the other hand, control cells treated with PLGA/PVA NP presented more than 85% viability at all concentrations, implying no toxic effect. Nevertheless, in cells treated with PLGA/PVA NP at 5 and 10 kGy, the increase in dead cells was more prominent in cells treated with higher concentrations (>60 µg/mL). We found significant differences between NP irradiated with gamma radiation and non-sterilized NP, obtaining more than 35% inhibition of cell proliferation at 150 and 200 µg/mL (47% and 66%, respectively). Finally, at 72 h PCL/PVA NP presented about 80% cell viability from 60 µg/mL with no statistical significance. We found a relative proliferation recovery in NP exposed to 10 kGy in gamma irradiation, while PLGA/PVA NP inhibited cell proliferation in a concentration-dependent manner, obtaining more than 40% of inhibition at higher concentrations of 150 and 200 µg/mL after 5kGy and 10 kGy of gamma irradiation (Figure 5C) with significant differences.

These results could be related to the fact that the irradiation sterilization process could modify the chemical structure and surface composition of the NP. Nevertheless, the use of cryoprotectors could minimize these changes. For PCL/PVA NP, the use of trehalose is recommended because it allows the morphology and mean particle size to be maintained [21]. On the other hand, for PLGA/PVA NP, the use of mannitol was more effective to preserve these physical properties (PDI and zeta potential). In this sense, the cryoprotector participation under the irradiation sterilization process has been corroborated [53]. Trehalose has been reported as an irradiation protector of substrates such as DNA by scavenging free radicals such as OH in a concentration-dependent manner [54]. For this reason, the use of 20% (*w*/*v*) of trehalose as cryprotector in the PCL/PVA NP systems did not affect the mean particle size. Derived from the irradiation process, the presence of sub-products could affect the interaction between cells and NP. However, the reduction in cell viability was only evidenced at the highest gamma irradiation dose and the highest concentration. Meanwhile, for PLGA/PVA NP systems, the mean particle size (and PDI) showed an important decrease under 5 and 10 kGy gamma irradiation doses. Likewise, cell viability was affected in a significant way. This higher effect could be attributed to a minor irradiation protection by mannitol and to the lower concentration used (3.5%, *w*/*v*). This cryoprotector has been reported as a radiation-sensitive material, so the presence of monomeric radicals of lactic or glycolic acid is highly probable [13,14,53,55,56]. The difference in the preservation of cryoprotectants between trehalose and mannitol to prevent cell damage can be attributed to a greater steric effect of the two glucose units present in trehalose instead of a short hydrocarbon chain of mannitol.

**Figure 6:**
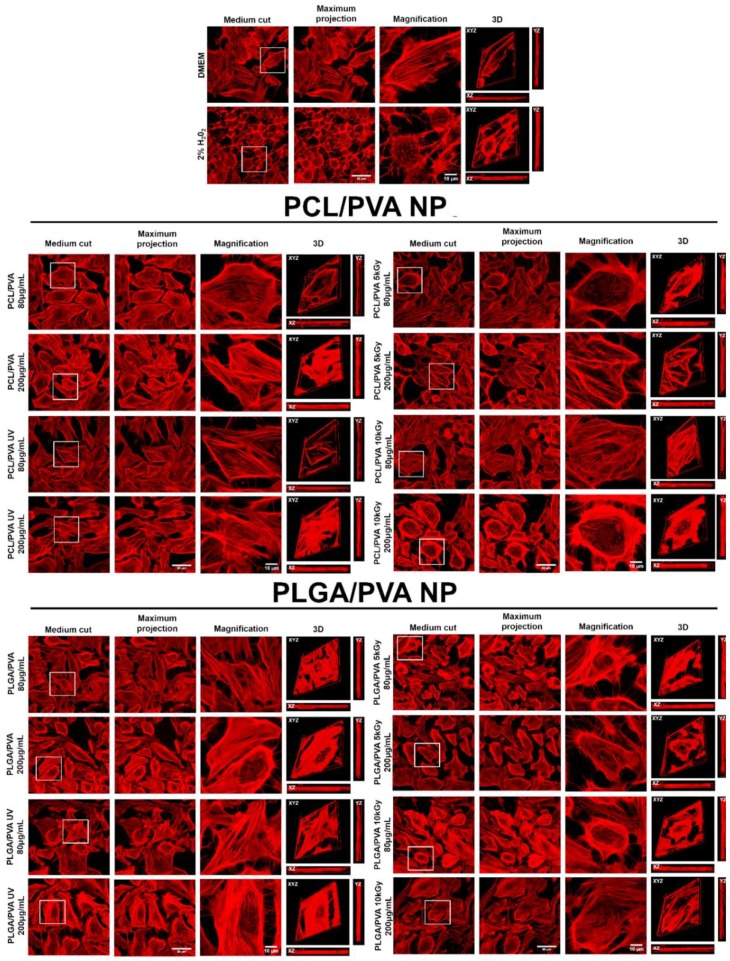
Phalloidin immunolocation in MIO-M1 cells exposed to 80 and 200 µg/mL of PCL/PVA and PLGA/PVA NP for 48 h. Upper: untreated cells with maintenance medium and control of cellular stress with treatment of 2% H_2_O_2_ are shown. Middle: MIO-M1 cells were treated with non-irradiated or irradiated PCL/PVA NP with UV light or gamma irradiation (5 or 10 kGys). Lower: MIO-M1 cells were treated with non-irradiated or irradiated PGLA/PVA NP with UV light or gamma irradiation (5 or 10 kGys). Scale bar = 10 μm. We performed microscopy visualization by medium cut, maximum projection magnification and 3D reconstruction.

To determine if NP induced morphological changes associated with a change in cytoskeleton, the location of actin was visualized by phalloidin, which is the main protein involved in the cytoskeleton’s filamentous network. Phalloidin immunostaining (Figure 6) showed that cells treated with 200 µg/mL of UV irradiated PCL/PVA NP did not have well-defined edges of the cell structure, and 200 µg/mL of 10kGy gamma-irradiated PCL/PVA NP showed very dense regions in the cellular periphery, an increase in the cell volume, and a cellular structure with fewer filaments. On the other hand, in cells treated with 5kGy gamma-irradiated PLGA/PVA NP, at both concentrations (80 µg/mL and 200 µg/mL), cellular structures were distant and smaller. Likewise, treatment with 200 µg/mL of 10kGy gamma irradiated PLGA/PVA NP to provoke an increase in the cellular area and the presence of less dense regions, principally in the center, a similar effect to that found in cell stress controls treated with H_2_O_2_. It is common for normal human fibroblasts to change their morphology from a spindle shape to an expanded, flattened, and irregular shape during the increase of reactive oxygen species under oxidative stress response that causes cellular senescence [57,58]. On the other hand, non-treated PCL/PVA NP and PLGA/PVA NP showed well-defined structures and a normal arrangement in the cytoskeleton structure, respectively. These results confirm that gamma radiation exposure generates greater alterations at the cellular level.

Since morphological changes are related to an alteration in the composition and distribution of proteins that constitute the cytoskeleton, we determined the effect of NP on actin reorganization. NP induced important changes in the reorganization of actin after gamma irradiation, with a maximum effect observed after 48 h of treatment in both NP treatments. The formation of free radicals could be the cause of an increase in oxidative stress and hence involvement in cell morphology. These results confirm that gamma radiation exposure generates more significant alterations at the cellular level.

These biological results could suggest that the UV sterilization is better for these systems, and also it is a terminal method. An advantage of UV sterilization is that it can be scaled to industrial levels. There are many industries where the use of UV light sterilization can provide a safe, effective solution, such as in the food and pharmaceutical industries, where UV lamps are used for irradiation. Ultraviolet light sterilization kills viruses, bacteria, yeast, and fungi in seconds and can also extend shelf life and nutritional value. Applications include packaging materials, conveyor belts, transport containers, working surfaces, countertops, and on liquid tanks and multiple vials.

Areas of opportunity for our work lie in exploring in detail the impact of radiations in the molecular weight of the excipients, the structural integrity of drug loading, the repercussions on the drug release profiles, as well as their effect on pharmacological activity. Therefore, we suggest these issues as crucial aspects in subsequent studies.

## 4. Conclusions

In this study, we demonstrated the feasibility of the radiation sterilization of PCL/PVA and PLGA/PVA NP. Microbiological monitoring revealed the presence of bacteria and fungi in the batch immediately after manufacturing and evidenced the efficiency of the sterilization process through UV and low doses of gamma irradiation to eradicate microorganisms. UV irradiation affected neither physicochemical parameters nor the viability of NP-treated cells until 200 µg/mL of NP, whereas gamma irradiation at low doses (5 and 10 kGy) slightly modified the mean particle size and zeta potential but not chemical properties. Effects on cell viability were observed at the highest NP concentration (200 µg/mL) with 10 kGy of gamma irradiation. A protective effect of the cryoprotectant trehalose was proposed to diminish the effects of radiation on cell viability. Meanwhile, UV and low gamma irradiation may become a useful sterilization process; the parameters of sterilization for other NP systems may require adjustment depending on the structure and components of each system. Future studies on loaded drugs NP are required to confirm the sterilization efficiency on drug release of different NP constitutions.

## Figures and Tables

**Table 1:** Size, polydispersity index (PDI), and zeta potential of PCL/PVA and PLGA/PVA NP. Data registered before and after sterilization.

Sample	Size (nm)	PDI	Zeta Potential (mV)
PCL/PVA NP	228.8 ± 11.60	0.050 ± 0.10	−14.47 ± 1.76
PCL/PVA NP UV	232.5 ± 5.70	0.040 ± 0.06	−14.07 ± 0.25
PCL/PVA NP 5 kGy	213.6 ± 2.21 *	0.080 ± 0.03	−18.53 ± 0.78 *
PCL/PVA NP 10 kGy	208.8 ± 1.37 **	0.110 ± 0.05 **	−22.90 ± 0.66 **
PLGA/PVA NP	243.1 ± 3.06	0.064 ± 0.02	−17.00 ± 0.17
PLGA/PVA NP UV	240.0 ± 1.55	0.070 ± 0.05	−18.16 ± 0.58
PLGA/PVA NP 5 kGy	209.6 ± 1.95 ***	0.046 ± 0.02	−17.00 ± 0.50
PLGA/PVA NP 10 kGy	217.0 ± 1.96 ***	0.028 ± 0.03 **	−17.50 ± 0.40

* *P* ≤ 0.1, ** *P* ≤ 0.01, and *** *P* ≤ 0.001 compared with no irradiated system.

**Table 2:** Main thermal events of the excipients and NP before and after sterilization by UV and gamma radiation recorded by differential scanning calorimetry.

Sample	T_1_ (°C)	T_2_ (°C)	T_3_ (°C)	T_4_ (°C)
PCL	67.5	-	-	-
PVA	195.5	-	-	-
D-(+)-trehalose dehydrate	100.0	212.0	-	-
PCL/PVA NP	56.0	99.5	211.0	-
PCL/PVA NP UV	56.7	99.7	212.0	-
PCL/PVA NP 5 kGy	54.8	97.8	130.0	212.0
PCL/PVA NP 10 kGy	55.8	98.5	130.0	212.0
PLGA	50.4	241.4	-	-
Mannitol	167.0	-	-	-
PLGA/PVA NP	165.3	-	-	-
PLGA/PVA NP UV	165.5	-	-	-
PLGA/PVA NP 5 kGy	165.1	-	-	-
PLGA/PVA NP 10 kGy	163.6	-	-	-

## Supplementary Materials

The following are available online at https://www.mdpi.com/1996-1944/13/5/1090/s1, Figure S1: Number of surviving microorganisms after exposure to UV irradiation. The systems were inoculated with 1 × 10^8^ CFU/mL of Escherechia coli, Staphylococcus aureus, and Candida albicans an exposure to different times of UV irradiation (A) PCL/PVA NP 1 µg in solid state, (B) PCL/PVA NP dispersed 40 µg/mL, (C) PLGA/PVA NP 1 µg in solid state, (D) PLGA/PVA NP dispersed 40 µg/mL, Figure S2: Morphology of nanoparticles before and after sterilization with UV and gamma irradiation. Images by Scanning Electron Microscopy (SEM). (A) PCL/PVA, (B) PCL/PVA UV, (C) PCL/PVA 5 kGy, (D) PCL/PVA 10kGy, (E) PLGA/PVA, (F) PLGA/PVA UV, (G) PLGA/PVA 5kGy, (H) PLGA/PVA 10 kGy. All magnifications are at 30,000×.
