# Supplementary material for: Effect of UV and Gamma Irradiation Sterilization Processes in the Properties of Different Polymeric Nanoparticles for Biomedical Applications
Source: Materials (Basel). 2020 Mar 1;13(5):1090. doi: 10.3390/ma13051090 (PMC7084644; doi:10.3390/ma13051090)
Supplement: Supplementary file 1 [file materials-13-01090-s001.pdf]

materials

Article

Effect of UV and Gamma Irradiation Sterilization Processes in the Properties of Different Polymeric Nanoparticles for Biomedical Applications

Y.S. Tapia-Guerrero [1,2,£], M.L. Del Prado-Audelo [3,4,£], F.V. Borbolla-Jiménez [2,5], D.M. Giraldo Gomez [6,7], I. García-Aguirre [8], C.A. Colín-Castro [2,9], J.A. Morales-González [1], G. Leyva-Gómez [4,*] and J.J. Magaña [1,2,10,*]

[1] Sección de Estudios de Posgrado e Investigación, Escuela Superior de Medicina, Instituto Politécnico Nacional, Plan de San Luis y Díaz Mirón, Ciudad de México, 11340, México; yessicasarai@gmail.com (Y.S.T.G.), jmorales101@yahoo.com.mx (J.A.M.G.)
[2] Laboratorio de Medicina Genómica, Departamento de Genética (CENIAQ), Instituto Nacional de Rehabilitación-Luis Guillermo Ibarra Ibarra (INR-LGII), Ciudad de México, 14389, México; fvbj@hotmail.com (F.V.B.J.), usedat@gmail.com (C.A.C.C.)
[3] Laboratorio de Tecnología Farmacéutica, Facultad de Estudios Superiores Cuautitlán, Universidad Nacional Autónoma de México, Cuautitlán Izcalli, Edo. de México, 54740, México; luisa.delpradoa@gmail.com
[4] Departamento de Farmacia, Facultad de Química, Universidad Nacional Autónoma de México, Ciudad Universitaria, Ciudad de México, 04510, México; gerardoleyva@hotmail.com
[5] Programa de Ciencias Biomédicas, Facultad de Medicina, Universidad Nacional Autónoma de México, Ciudad de México,04510, México.
[6] Departamento de Biología Celular y Tisular, Facultad de Medicina, Universidad Nacional Autónoma de México (UNAM), Edificio "A" 3er piso, Circuito Interior, Avenida Universidad 3000, Ciudad Universitaria, Coyoacán, 04510 Ciudad de México, México; davidgiraldo@comunidad.unam.mx
[7] Unidad de Microscopía, Facultad de Medicina, Universidad Nacional Autónoma de México (UNAM), Edificio "A" planta baja, Circuito Interior, Avenida Universidad 3000, Ciudad Universitaria, Coyoacán, 04510 Ciudad de México, México.
[8] Departamento de Genética y Biología Molecular, Centro de Investigación y de Estudios Avanzados (CINVESTAV-IPN), Ciudad de México, 07360, México; ian.garcia@cinvestav.mx
[9] Departamento de Infectología, (CENIAQ), Instituto Nacional de Rehabilitación-Luis Guillermo Ibarra Ibarra, Ciudad de México (CDMX), 14389, México.
[10] Escuela de Ingeniería, Departamento de Biotecnología, Instituto Tecnológico y de Estudios Superiores de Monterrey-Campus Ciudad de México, 14380, México.
[£] These authors equally contributed to the work.
[*] Correspondence: gerardoleyva@hotmail.com (G.L.G.); Tel.: (+52) (55) 5622 3899; and jmagaña@inr.gob.mx; maganasm@hotmail.com (J.J.M.); Tel.: (+52) (55) 5999 1000

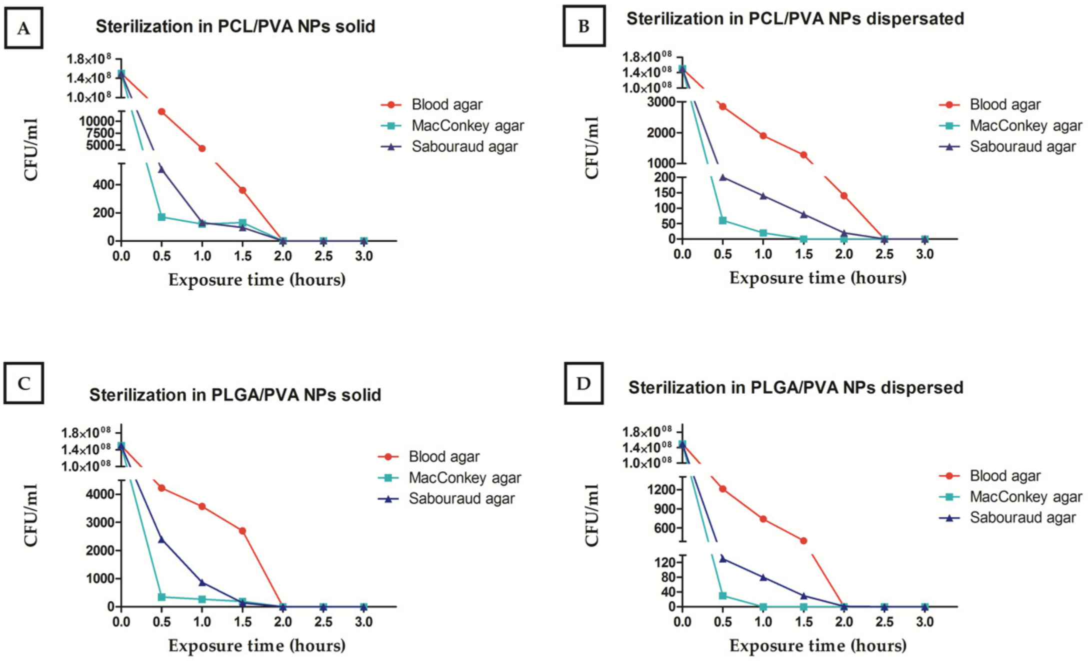

Figure 1. Number of surviving microorganisms after exposure to UV irradiation. The systems were inoculated with 1 × 10^8 CFU/mL of Escherechia coli, Staphylococcus aureus, and Candida albicans an exposure to different times of UV irradiation (**A**) PCL/PVA NP 1 µg in solid state, (**B**) PCL/PVA NP dispersed 40 µg/mL, (**C**) PLGA/PVA NP 1 µg in solid state, (**D**) PLGA/PVA NP dispersed 40 µg/mL.

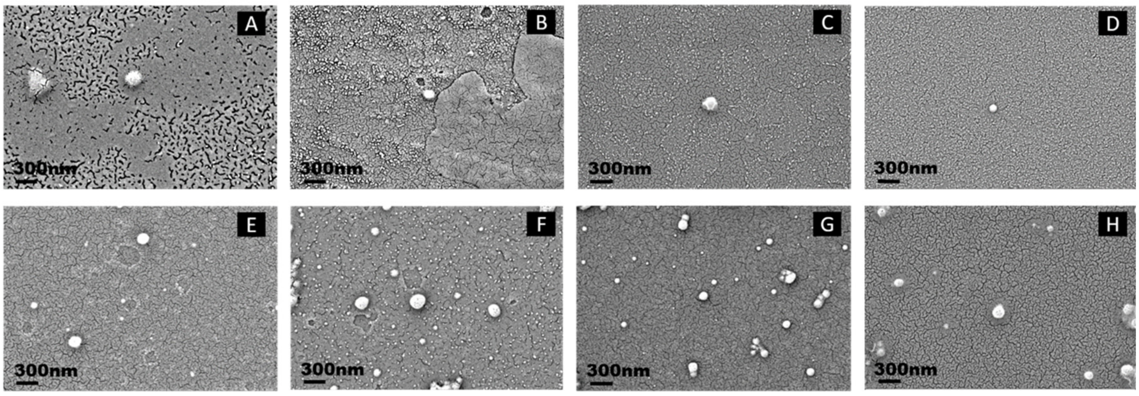

Figure 2. Morphology of nanoparticles before and after sterilization with UV and gamma irradiation. Images by Scanning Electron Microscopy (SEM). (A) PCL/PVA, (B) PCL/PVA UV, (C) PCL/PVA 5 kGy, (D) PCL/PVA 10kGy, (E) PLGA/PVA, (F) PLGA/PVA UV, (G) PLGA/PVA 5kGy, (H) PLGA/PVA 10 kGy. All magnifications are at 30,000×.
